# Antimicrobial Activity of Bee-Collected Pollen and Beebread: State of the Art and Future Perspectives

**DOI:** 10.3390/antibiotics9110811

**Published:** 2020-11-14

**Authors:** Nikos Asoutis Didaras, Katerina Karatasou, Tilemachos G Dimitriou, Grigoris D. Amoutzias, Dimitris Mossialos

**Affiliations:** 1 Microbial Biotechnology-Molecular Bacteriology-Virology Laboratory, Department of Biochemistry & Biotechnology, University of Thessaly, 41500 Volos, Greece; didasout@yahoo.gr (N.A.D.); tidimitr@bio.uth.gr (T.G.D.); 2 Apicultural Centre of Larissa, Federation of Greek Beekeepers Associations, 41500 Larissa, Greece; omse@otenet.gr; 3 Bioinformatics Laboratory, Department of Biochemistry & Biotechnology, University of Thessaly, 41500 Volos, Greece; amoutzias@bio.uth.gr

**Keywords:** bee products, bee collected pollen, beebread, antimicrobial activity, microbiome, functional food, antibiotic resistance

## Abstract

Bee-collected pollen (BCP) is a well-known functional food. Honey bees process the collected pollen and store it in the hive, inside the comb cells. The processed pollen is called bee- bread or ambrosia and it is the main source of proteins, lipids, vitamins, macro-and micro-elements in honey bee nutrition. During storage, beebread undergoes solid state fermentation which preserves it and increases the bioavailability of nutrients. Research on beebread has been rather limited until now. In recent years, there is an increasing interest regarding the antimicrobial properties of BCP and beebread, due to emerging antimicrobial resistance by pathogens. Both BCP and beebread exhibit antimicrobial properties against diverse pathogens, like bacteria and fungi. As is the case with other bee products, lack of antimicrobial resistance might be attributed to the synergy of more than one antimicrobial compounds within BCP and beebread. Furthermore, BCP and bee bread exert targeted activity against pathogens and affect the host microbiome in a prebiotic manner. This review aims to present up to date research findings regarding these aspects as well as to discuss current challenges and future perspectives in the field.

## 1. Introduction

Honey bee products (honey, bee collected pollen, beebread, royal jelly, propolis, bee venom, beeswax) are widely used since ancient times as traditional remedies. Nowadays there is an increasing interest regarding honeybee products, their bioactivity and implementation in alternative medicine and apitherapy.

Honey was the only sweet and energy rich food available to early Hominidae. Therefore, it is speculated by some anthropologists that honey was one of the main environmental factors contributing to accelerated human brain evolution [1]. Honey exerts antibacterial [2,3,4] and antifungal [5] activity in vitro, but these properties are highly variable among different honey types [6]. These biological properties are attributed to physical and chemical factors such as low pH, high sugar content (high osmolality), hydrogen peroxide production from glucose oxidase activation and additionally to other chemical compounds such as methylglyoxal, 3-phenyllactic acid (PLA), bee defensin, Major Royal Jelly Proteins (MRJPs) and bacteriocins [7]. Biological factors such as honey microbiome may contribute to these properties though its role is not fully elucidated. Honey exerts an anti-inflammatory action that can quickly ease pain and inflammation, thus contributing to wound healing [6]. It also exhibits variable antioxidant properties depending on its botanical origin. Different types of honey are reported to exhibit anticancer properties [8,9,10,11,12]. Furthermore, honey is considered as a prebiotic food positively affecting the human microbiome and well-being [13,14,15].

Propolis consists mainly of resins that honey bees gather from the exudate found in tree buds, sap flows or other botanical sources which then are mixed with bee saliva and beeswax inside the hive. It is used to seal cracks and narrow openings, to protect the hive from invaders or cold weather conditions. Still, the most important use is in sanitization, since honey bees coat the nest walls and the comb cells that are going to be used either for brood rearing or food storage before use with propolis. The use of propolis by the bee colony might be considered as an agent of social immunity [16]. Propolis exhibits a variety of biological properties including antibacterial, antifungal, antiviral as well as antioxidant, anti-inflammatory and anti-cancer properties [17,18].

Royal jelly (RJ) is produced by the hypopharyngeal glands of very young bees called nurse bees. It is the only food for the queen bee. Nurse bees feed with RJ the young larvae, the young drones and the forager bees of the colony [19]. Royal jelly is known for its anti-inflammatory and immunomodulatory activity [20]. Furthermore, royal jelly components such as *trans*-10-hydroxy-2-decenoic acid, royalisin, Major Royal Jelly Proteins (MRJPs) and their cleavage products jelleines [21,22], as well as glucose oxidase contribute to the antimicrobial activity against bacteria, fungi and viruses [23,24].

Bee venom (BV) is known to exert antimicrobial, anti-inflammatory [25,26], neuroprotective [27], and anticancer [28] effects. The antimicrobial properties of BV have been shown in vitro and in vivo against bacteria, viruses and fungi [29]. Synergistic therapeutic interactions of BV with antibiotics have also been reported [30]. In addition BV has shown trypanocidal activity [31] and it appears to prevent neuronal cell death caused by prion peptides [32].

Beeswax has also been reported to exert antimicrobial activity [33] but the related literature is very limited.

Bee-collected pollen (BCP) and beebread (BB) are appreciated mainly for their high nutritional value. Both products are rich in proteins, essential amino acids, sugars, fatty acids (including ω-3 and ω-6 fatty acids), vitamins, macro and microelements. In addition, they are regarded as functional foods because they are rich in polyphenolic compounds and exhibit significant antioxidant properties. They also contain a wide variety of other health promoting compounds present in functional foods, such as prebiotics, probiotics, fibre, lignans, triterpenes, carotenoids, bioactive peptides and organic acids [34,35,36,37]. Supplementation of livestock and poultry diets with BCP enhanced growth performance, immunity responses and carcass quality and was considered an excellent substitute of antibiotics [38]. In many countries they are implemented in apitherapy or other types of complementary medicine for a large variety of impaired health conditions [36,39,40].

Pollen is the only source of protein and lipids, and the main source of macro and microelements for the honey bee colony, while nectar or honeydew (processed and stored as honey) is the main source of carbohydrates [41]. Smaller amounts of pollen are stored in the colony compared to honey and they are quickly depleted during periods of little or no forage [42,43]. A colony can collect as much as 10–26 kg of pollen per year [44].

The term “beebread” refers to the collected pollen that is processed by the bees, fermented and stored in the hive. Honey bee foragers unload the pollen pellets they carry back to the hive directly into the empty cells of the comb, between the brood cells and the honey cells, thus forming a typical band of beebread available for immediate consumption by the nurse bees. Foragers utilize regurgitated nectar to hold the pollen grains together thus forming the pellet. Then, the middle aged bees, add honey and glandular secretions and pack it tightly inside the comb cell, pushing with their heads. It is assumed that fermentation of this mixture and possibly the predigestion of pollen grains by added bee enzymes as well as the beebread microbiota preserve bee bread and promote its nutritional value [45]. The microbial communities of bee bread could produce their own antimicrobial compounds thus further contributing to its bioactivity.

BCP demonstrates at least in vitro, antibacterial, and antifungal activity [46,47,48]. There are significantly less data concerning bee bread but nevertheless it has been established that it exerts significant antimicrobial activity [35,49,50].

Although most antimicrobial compounds of bee products have been identified (Table 1), their mode of action is still not fully described. Similarly, the contribution of microbiome in the antimicrobial activity exerted by bee products remains to be elucidated in detail.

This review aspires to present state of the art research findings on the antimicrobial activity of pollen and bee bread as well as current challenges and future perspectives.

## 2. Physicochemical Composition of Bee-Collected Pollen (BCP) and Beebread (BB)

The chemical composition of bee collected pollen, depends strongly on botanical and geographic origin, climate, soil type, season and weather conditions during collection, as well as on bee race and even beekeeping management [70,71]. Different studies reviewed by Campos et al. [72], have shown that bees are very selective when gathering pollen and that the bulk of the collected pollen comes from few plant species. The identification of botanical origin of both BCP and BB is of paramount importance since their biological, nutritional, antioxidant and antibacterial properties are directly related to their composition [73,74].

It should be noted that crude nutrient measurements in BCP and BB cannot accurately determine their nutritional or antioxidant value for honey bees or human consumers. The bioavailability of nutrients is of importance, especially considering the fact that they are encapsulated inside the hard to crack pollen grains. Pollen grain outer walls consist of two layers: the outermost (exine) is made of an enduring biopolymer called sporopollenin while the inner surface (intine) is made of elastic cellulose microfibrils and pectin [75]. For this reason it might be more appropriate to assess the effect of BCP or BB diets on honey bees and humans alike [76].

It is suggested that the breach of pollen grains occurs naturally during BCP fermentation catalyzed by bee enzymes and microorganisms (bacteria, molds and yeasts). Beebread is more nutritious for the bees compared to BCP [77,78].

The beebread microbiome plays a pivotal role in nutrition and development of honey bees [79,80]. Several studies have demonstrated that BB microorganisms facilitate the enzymatic pre-digestion of pollen grains [45,48,79], thus increasing nutrient content and digestibility [81,82]. Vásquez and Olofsson suggested that lactic acid bacteria from the bee honey stomach are involved in BB fermentation and might be responsible for improving its nutritional value by producing vitamins [80].

On the other hand, Anderson et al. totally disagreed with the idea that microbial pre-digestion of pollen or nutrient conversion takes place during BB maturation [83]. Additionally, Nichholson et al. [84], fed caged young workers fresh and stored pollen. He observed that pollen digestibility was high and storage of sunflower pollen did not increase its digestibility thus suggesting that pollen storage does not confer obvious benefits to honey bees.

The fact that bees use stored beebread in early spring to feed the first brood after overwintering, is in favor of its higher nutritional value. Furthermore, Beutler and Opfinger observed that bees fed on beebread lived longer compared to bees fed on pollen collected from traps [85]. However, Carrol et al. observed that two to four day-old pollen was significantly more likely to be consumed, instead of seven days old pollen [86]. Furthermore, young adult workers reared for 7 days on 1d-, 5d-, or 10d-old stored pollen demonstrated no difference in body mass, hindgut fecal accumulation or hypopharyngeal gland protein titers, suggesting that pollen age is irrelevant to its nutritional value [86].

Whether pollen fermentation or/and storage affects pollen nutritional value and its palatability for bees remains controversial up to date. Nevertheless, a recent study presents a new, interesting concept: when larval bees consume beebread, they consume both microbial and plant biomass, assimilating microbial amino acids as well as those of plant origin (pollen), which makes bees omnivorous rather than herbivorous [87]. Dharampal et al. also observed that pollen microbes provide substantial nutrition to mason bee larvae [82]. They suggested that microbial communities associated with aged pollen are central to bee health, not only as nutritional mutualists, but as a major dietary component as well.

Moisture varies from 21.7–27% for BCP, whereas a wider range is observed for BB moisture values: 11.41–28% [88,89]. Protein content was determined between 7 and 40% g/100 g of BCP and 14–37% for BB [90,91,92]. According to Nagai et al. [92], bee bread contains large quantities (20%) of easily assimilated proteins and all human essential amino acids. These include glutamic acid, aspartic acid, proline (the most abundant amino acid), arginine, valine, histidine, leucine, isoleucine, lysine, methionine, tryptophan, phenylalanine, threonine, cysteine, tyrosine, alanine, glycine and serine [93]. Enzymes present in beebread include amylase, invertase, phosphatase transferases and glucose-oxidase. Enzymatic cofactors are also found in BB, including biotine, glutathione, NAD, and some nucleotides [94]. Anđelković et al. [95], compared BCP and BB samples derived from the same bee colonies. They found that the content of crude protein was increased by 19.91% in BB compared to BCP.

Carbohydrates varied between 24–60 g/100 g of BCP and 24–35 g/100 g of BB [92,93,96]. Bakour et al. [35], measured carbohydrates to reach 74.82 ± 0.04 g/100 g bee bread and the main identified free sugars were fructose (11.8 ± 0.6 g/100 g BB), glucose (5.7 ± 0.4 g/100 g BB) and trehalose (0.92 ± 0.01 g/100 g BB). BB contains less starch compared to pollen [88]. According to Roulston and Cane [76], starch content in pollen varied from 0–22 g/100 g. Anđelković et al. [95], determined the cellulose content in BCP (3.7 g/100 g) and BB (2.7 g/100 g). During pollen fermentation which takes place in the comb cells, carbohydrates are metabolized by *Lactobacillus* spp. and lactic acid is produced up to 3.2% [92,93]. BCP pH ranges from 3.8–6.3 and BB pH from 3.8–4.3 [88,90,93].

Lipids ranged from 1–13 g/100 g in BCP [97] and 6–13 g/100 g for BB [92]. Bakour et al. [35], found the total lipid content of beebread to be 1.90 ± 0.06 g/100 g BB. Kaplan et al. [89] estimated the lipid content in beebread between 5.93 g/100 g BB and 11.55 g/100 g BB. Zuluaga et al. [91], analyzed fifteen BB samples and they determined the lipid content between 1.65 to 5.50 g/100 g (mean 3.40 ± 1.08 g/100 g). Observed differences, could be attributed to botanical origin of the BB. Anđelković et al. [95], reported that the content of crude fat in BB is increased by 4.47% compared to BCP.

At least 14 fatty acids (FAs) were identified in BCP samples from Romania, Poland, South Korea and China, the most abundant being α-linolenic, palmitic and linoleic [74,98]. Kaplan et al. [89], identified 37 FAs in 8 BB samples from Turkey and found the unsaturated to saturated ratio ranged between 1.38 and 2.39, indicating that BB is a good source of unsaturated fatty acids.

Fatty acids are important nutrients playing a major role in honeybee fertility and health [99]. Polyunsaturated fatty acids, especially omega-3 PUFAs, are important in human nutrition and health as well because they reduce triglyceride and cholesterol levels in blood [100]. Furthermore, they exhibit anti-inflammatory and antithrombotic properties [101]. Omega-3 PUFAs also play a role in cancer prevention and treatment [102]. Fatty acids are known antimicrobial agents [67,68].

Organic acids are also found in BCP like acetic, citric, lactic, malic, oxalic, tartaric, succinic acid, gluconic acid being the most abundant [36].

BCP contains water-soluble vitamins, such as B-complex vitamins, vitamin C, rutin and inositol as well as fat soluble vitamins, including A (mainly b-carotene), E and D [103,104]. BB is rich in B-complex vitamins and vitamin K, which is not present in fresh pollen [105,106]. According to Hryniewick et al. [107], bee bread is rich in α-tocopherol (80 ± 30 μg/g) and contains relatively small amounts (11.5 ± 0.3 μg/g) of coenzyme Q10, one of the most important cellular antioxidants [108].

Moreover, pollen contains over 25 different micro- and macroelements such as iron, calcium, phosphorus, potassium, copper, zinc, selenium, and magnesium [40]. The predominant minerals in BB were potassium, followed by phosphorus, calcium, magnesium, iron, sodium, zinc and manganese [35]. Dry matter-based ash in BCP was found to be 2.5 ± 0.4 g [90]. Ash in BB ranged between 1.9 and 2.54% [89], and 3.32 ± 0.08% [35]. Anđelković et al. [95], reported that the ash content was increased by 7.54% in BB compared to BCP. Furthermore, calcium, potassium, phosphorus, magnesium and iron content was increased compared to BCP while zinc and manganese decreased. This could be attributed to microbial metabolism which takes place during fermentation of pollen into beebread [109,110].

Vanderplanck et al. [111], identified sterol compounds in BCP derived from many plant species (*Calluna vulgaris* L. Hull, *Cistus* spp., *Cytisus scoparius* (L.) Link, *Salix caprea* L. and *Sorbus aucuparia* L.). Major phytosterols included β-sitosterol and δ5-avenasterol. Significant amounts of δ7-avenasterol (in *C. vulgaris*, 20.23%) and 24-methylenecholesterol/campesterol fraction (*S. aucuparia* L., 84.07%) were measured in several pollen samples. Phytosterols exhibit therapeutic potential: they induce cell apoptosis, reduce cholesterol levels and exert cardioprotective and anti-inflammatory activity [112,113,114].

Polyphenol compounds provide the distinct color of pollen grains (yellow, brown, black, red, purple, etc.) and their characteristic organoleptic feature is bitter taste [115]. Polyphenols, as effective antioxidants, contribute to cancer prevention [116,117,118], and treatment of diabetes [119,120], atherosclerosis [121] and cardiovascular diseases [122], autoimmune diseases [123] and even dementia [120,124]. They also exhibit strong antimicrobial activity [62,63]. Polyphenol compounds in pollen are chemically characterized as flavonoids and phenolic acids. Several authors tested different extraction solvents, such as methanol, ethanol, hexane or water, in order to evaluate their effect on bioactive compound concentration as well as bioactive properties of BCP and BB [71].

In BCP samples of diverse botanical origin, tricetin, luteolin, selagin, myricetin, rhamnetin, isorhamnetin, isoquercetin, catechin, narigenin, apigenin, quercetin and kaempferol, are the most identified flavonoids. The latter two and their glycosidic forms are the most abundant [36,125,126]. Other flavonoids in bee pollen are leucoanthocyanidins and catechins [127].

Phenolic acids present in BCP include syringic, coumaric, sinapic, ferulic, cinnamic, chlorogenic, gallic and abscisic acid [126,127,128] caffeic acids [129], as well as hydroxycinnamic, ortho-coumaric and para-coumaric acids [128,130,131].

Relevant publications regarding BB are less numerous. Isidorov et al. [34], using GC-MS, reported naringenin, kaempferol, apigenin, isorhamnetin, and quercetin as the main flavonoids and *p*-coumaric acid as the main phenolic acid in BB samples from Latvia, Russia and Poland. Markiewicz-Zukowska et al. [116], analyzed three bee bread samples from Poland, by GC-MS and determined kaempferol and apigenin as the main flavonoids. Tavdidishvili et al. [132], detected the presence of 12–15 flavonoids by HPLC in Georgian beebread samples; the most abundant were naringin, rutin and quercetin (20% of total flavonoid content). Sobral et al. [117] analyzed six beebread samples from Portugal and they reported that the main phenolic compounds were quercetin, kaempferol, myricetin, isorhamnetin and herbacetin glycoside derivatives. Bakour et al. [35], analyzed the phenolic composition of one Moroccan beebread sample and reported the presence of thirteen polyphenolic compounds, the most abundant being quercetin, kaempferol, isorhamnetin, and methylherbacetrin glycoside derivatives.

Urcan et al. [71], conclude that literature data show wide variability regarding the content of phenolic compounds in BB presumably due to diverse botanical origin. Nevertheless, variability could also be attributed to different extraction and quantification methods that have been implemented in various studies.

A recent study published by Urcan et al. [133], comparing pollen, BCP and BB samples from Romania and India, demonstrated that the phenolic profile of BB is identical to that of hand collected flora pollen as well as bee collected pollen. They reported that in spite of biochemical changes that might take place during BCP fermentation into bee bread, the phenolic compounds were not affected. In addition, these authors reported that factors such as soil and climate might not influence phenolic compounds in studied samples. A most recent study demonstrated that the total phenolic and flavonoid content as well as the radical scavenging activity increased by 1.27–2.40 fold in polyfloral BCP, following either spontaneous fermentation or fermentation using *Lactococcus lactis* and *Lactobacillus rhamnosus* as inoculum [48].

In conclusion, BCP and BB chemical composition varies a lot depending mostly on the botanical origin. Wide variations both in composition and concentration of constituents in BCP and BB could be attributed to possible pre-digestion of pollen grains during BCP fermentation to BB. Furthermore, vitamins, amino acids and other nutrients might be produced by BB microbiome. There are indications that BB preservation is not due to a mere ensilage but more studies regarding this aspect should be conducted in the future.

Sampling is very important, especially when samples are taken from commercially available products and not directly from the hive. Storage conditions may deteriorate sample quality. Further comparative studies, using BCP and BB samples taken from the same hive at the same time, could unequivocally demonstrate to what extent BB is nutritionally more enhanced than BCP.

## 3. BCP and BB Microbiome

It has been suggested that bees evolved from ancient carnivorous wasps, during the Middle to Late Cretaceous by acquiring a specialized pollen-digesting gut microbiome which allowed them to feed on herbivorous diet [134]. These ancestral bacterial communities are no longer part of *Apis mellifera* core gut microbiome [135]. Modern era *A. mellifera* adult workers host a characteristic gut microbiome of nine distinct bacterial phylotypes, accounting for more than 95% of total bacterial diversity. These findings have been reported in several studies, analyzing samples acquired from different environments, continents and host genotypes [135,136,137,138]. Beebread, the major brood food source, lacks bacterial phylotypes which are commonly present in adult worker microbiome [139]. Saraiva et al. [79], assessed the diversity and community structure of bacteria and archaea in Africanized honeybee guts and beebread and observed that only 7% of species-level taxa were shared between bee gut and beebread.

Health status of individual honey bees and the colony as a whole, depends on symbiotic relationships with gut microbiome [140,141,142]. Gut microbiome is involved in pollen digestion, carbohydrate utilization and immune response [143,144]. In that aspect, Rokop et al. [145], used culture-based methodology and molecular taxonomy (sequencing of 16S rRNA gene) to characterize lactic acid bacteria (LAB) communities present across bee hive environments. In the process, they identified interactions between non-core bacterial members (*Fructobacillus* spp. and Lactobacillaceae) and honey bee-specific core members. Both *Fructobacillus* spp. and Lactobacillaceae colonize brood cells, beebread and nectar and might be pioneering species, establishing an environment conducive to the inoculation by honey bee core bacteria. Co-culture assays have demonstrated that these bacteria promote the growth of honey bee specific bacterial species. Interestingly, *Fructobacillus* spp. by products in spent medium supported the growth of the Firm-5 honey bee specific clade in vitro. Metabolic characterization of *Fructobacillus* using carbohydrate utilization assays revealed that this strain is capable of utilizing simple sugars such as fructose and glucose as well as the complex plant carbohydrate lignin.

Manirajan et al. [146], investigated the bacterial abundance, diversity and community structure in birch, rye, rape and autumn crocus pollen by cultivation, sequencing and microscopy. Proteobacteria was the most abundant phylum in all pollen species, followed by Actinobacteria, Acidobacteria and Firmicutes. According to this study, microbial communities of insect-pollinated plant species were more similar among them compared to wind-pollinated species, suggesting that insects shape microbial communities. Ushio et al. [147], hypothesized that part of the microbial community on a flower’s surface is transferred there from insect body surfaces and that this community can provide information to identify potential pollinator insects of that plant. In order to test this hypothesis, laboratory experiments demonstrated that the microbial community composition on a flower surface indeed changed after contact with an insect, suggesting that microbes are transferred from the insect to the flower.

Gilliam analyzed pollen, BCP and BB samples of different age, all derived from the same plant species and demonstrated that changes in the biochemical and microbiological composition of flower pollen start as soon as the bee collects it [45].

Furthermore, Gilliam reported that the dominant microbes in pollen and BB were fungi (55% of total pollen isolates and 85% of BB total isolates). Bacteria were up to 49% in pollen, decreased to 28% in BCP and comprised only 4% of BB microbiota. *Bacillus* spp represented 2% of pollen microbes, 20% of BCP microbes and 11% of BB microbes. *Bacillus subtillis*, the only *Bacillus* associated with floral pollen, was more abundant in BCP and up to 1 week-old BB [148]. Regarding molds, Penicillia, Mucorales and Aspergilli were the most abundant.

Vasquez and Olofsson [80], investigated the presence of LABs, previously isolated from bee honey stomach, in samples of BCP, two week –old and two month- old BB. LAB community comprised twelve species belonging to *Lactobacillus* and *Bifidobacterium* [80,138]. Most of these species were viable in BCB and the two week -old BB but not in older BB samples. Authors suggested that LABs are actually inoculated to BCP by bees which regurgitate nectar. LABs could be considered as a starter culture in BCP fermentation. Furthermore, antimicrobial compounds produced by LABs could preserve BB and protect honeybees from diseases [80].

Mattila et al. [149], assessed the bacterial diversity in bee gut and BB samples. *Succinivibrio*, *Oenococcus*, *Paralactobacillus*, and *Bifidobacterium* were the predominant genera found in bee gut samples accounting for more than 67% of its bacterial communities. Two bacterial phyla (Proteobacteria and Firmicutes) were identified in beebread samples whereas the most abundant genera were *Oenococcus*, followed by *Paralactobacillus*, *Shimazuella* and *Saccharibacter*. Out of 18 identified species in BB, 17 were facultative or obligate anaerobes. These species included many LABs such as *Oenoccoccus*, *Paralactobacillus*, *Bifidobacterium* as well as *Enterobacteria* (*Enterobacter*, *Escherichia*, *Shigella*, *Klebsiella* and *Serratia*). The authors suggested that BB is stratified with respect to oxygen tension, which may allow it to support a range of bacteria, from aerobes to strict anaerobes.

Anderson et al. [83], performed behavioral assays, bacterial count assays, microscopy and pyrosequencing of 16S rRNA gene to elucidate the structure of bacterial communities in BCP and BB samples. They found that newly collected pollen contained few bacteria and these numbers declined in stored pollen after 96 h. A small subset of bacteria were present in both newly collected and stored pollen regardless of season, indicating associations with bacteria present in the hive. Bacterial communities of BB were highly dissimilar to those found in the honey bee gut or those found on the body surface of foraging bees. Bee core gut bacteria were found at a greater relative abundance in newly collected than stored pollen. BB samples had three times the number of unique bacterial sequences compared to newly collected pollen, among which were *Bradyrhizobiaceae*, *Xanthomonadaceae*, *Enterobacteriaceae*, *Rhodobacterales*, *Pseudomonadales*, *Bacteriodetes* and many groups of Actinobacteria. At least two acid resistant and osmotolerant microbes, *Lactobacillus kunkeei* and *Parasaccharibacter apium*, were present in relative high proportion in BB. In this study Anderson et al. implemented several methods to demonstrate that no nutrient conversion or pre-digestion by microbes takes place during BCP transformation to BB which in turn suggests that added honey, nectar, bee secretions and antimicrobial compounds of pollen itself could preserve BB.

In a study by Asama et al. [150], *Lactobacillus* was found to be the dominant genus both in BB (83.9%) and BCP (74.6%) samples from Japan, followed by *Burkholderia* (2.1%) in BB and *Gluconobacter* (3.4%) and *Paenibacillus* (2.1%) in BCP samples. *Lactobacillus kunkeei* was the most abundant species of all *Lactobacillus* spp, (99.5% in BB and 98.6% in BCP) as well as in honey, royal jelly and bee honey stomach.

Donkersley et al. [151], using Next Generation Sequencing (Illumina MiSeq) and denaturing gradient gel electrophoresis (DGGE), assessed the microbial diversity of BB samples from North West England. Analysis revealed 24 bacterial phyla, Proteobacteria being the most dominant phylum. All BB samples included on average 13 bacterial phyla, the most abundant being Bacteroidetes, Firmicutes, α-Proteobacteria, β-Proteobacteria, and γ-Proteobacteria. *Enterobacteriaceae* was the most abundant family found in BB. In each BB sample, 96 bacterial genera were found on average and the five most common genera were *Pseudomonas* (32.4%), *Arsenophonus* (13.0%), *Lactobacillus* (8.2%), *Erwinia* (7.7%) and *Acinetobacter* (5.2%). Eleven genera were found in all samples, including *Pseudomonas*, *Arsenophonus*, *Orbus*, *Lactobacillus*, *Erwinia* and *Acinetobacter*, suggesting that these bacterial genera might be members of core BB microbiome. This study also correlated the bacterial diversity observed in BB with environmental factors, demonstrating that change of land use may have an indirect detrimental effect on BB microbiome.

Di Cagno et al. [152], investigated the structure of lactic acid bacteria communities in ivy flower pollen (FP), BCP, BB and honeybee gut. They demonstrated that the high microbial diversity of FP and fresh BCP was significantly reduced in BB. Most LAB species disappeared during BB maturation and in long stored BB. *Lactobacillus kunkeei* and *Fructobacillus fructosus* were the dominant species which were also abundant in bee honey stomach. Authors attempted to emulate the spontaneous fermentation of BB, inoculating BCP with selected *L. kunkeei* strains and *Hanseniaspora uvarum* AN8Y27B. They demonstrated an increase in digestibility as well as bioavailability of nutrients and bioactive compounds naturally occurring in BCP. Fermented BCP by selected mixed starters contained higher concentrations of peptides, free amino acids and free phenolics. Moreover, it has shown higher in vitro protein digestibility compared with spontaneously fermented BCP. This study highlighted the close relationship between lactic acid bacteria and yeasts during BCP fermentation.

Disayathanoowat et al. [153], investigated and compared both bacterial and fungal communities in BCP and BB of two honeybee species, *Apis mellifera* and *Apis cerana*. In this study, BCP was collected directly from honey bee legs. BB samples were collected after 48 and 72 h from the same comb cells. DNA was extracted from the samples and then, the 16S rRNA gene V3-V4 region as well as fungal ITS1-ITS2 region were amplified and sequenced using the Illumina MiSeq platform. Analysis showed a significant difference in the average number of both bacterial and fungal sequences in BB between the two honeybee species. Although the two species utilized different floral sources, they displayed similar core microbial communities albeit a difference in abundance. The most abundant bacterial phylum in all combined samples was Proteobacteria (92.12%), followed by a small population of Firmicutes (6.66%). In BCP from both species, Enterobacteriaceae were more abundant. However, the number of bacteria significantly decreased in hive-stored BB in less than 72 h. Initially, *Escherichia-Shiga* was the most abundant bacterial genus but then its proportion decreased to less than 10% of total bacteria operating taxonomic units (OTUs) in all BB samples. A similar pattern was observed for *Pseudomonas* and *Paracoccus*. In pollen collected by *A. cerana*, *Rosenbergeilla* and *Buttiauxella* genera were more abundant while in pollen collected by *A. mellifera Paracoccus* and core bee gut bacteria (*Bifidobacterium*, *Gilliamella*) were higher. The dominant fungal phyla, were Ascomycota (93.55%), followed by Basidiomycota (5.65%). *Cladosporium* that belongs to Ascomycota was the most abundant genus (52.20%). Only one Basidiomycete yeast genus, *Rhodosporidium*, accounted for 2.65% of the total microbial community. In this study it was demonstrated that as pH gets lower in BB, the same happens to bacterial populations while the fungal populations remain stable. *Cladosporium* remained the dominant fungal genus, inside the comb cells. According to authors, this particular filamentous fungus could help preserve pollen by releasing organic acids, along with other filamentous fungi that might inhibit both commensal/contaminant bacteria and the growth of pathogens.

## 4. Methodology to Study BCP and BB Antimicrobial Activity

BCP is more often collected using pollen traps adjusted at the entrance of bee hives or inside (bottom/top) of the bee hive. In some cases BCP has been collected directly from bee’s hind legs thus avoiding microbial contamination. Moreover, pollen has been directly collected from flowers to compare with BCP.

BCP samples after collection are usually stored in deep freeze (−18 °C) or are partly dried and then stored below 4 °C, or dried and stored in a cool dark place. Dried BCP compared to fresh BCP is less inhibitory against bacteria [50].

BB is extracted directly from the honey bee comb. It is difficult to estimate the actual age of BB inside the hive, because honey bees feed on it daily and may or may not replenish it once consumed. For sampling purpose, it is necessary to isolate a framing comb or a part of it inside the hive in order to prevent bees from storing fresh pollen in comb cells [153].

Various solvents used for extraction are presented in Table 2. Methanol and ethanol are most often used, followed by water. Used solvents may exhibit specific chemical affinity towards active substances. For example, water could extract more efficiently flavonoids such as quercetin and kaempferol glycosides [46].

Depending on the used solvent, extracts may show variable antimicrobial activity even against the same bacterial strain. Additionally, the use of different solvent concentrations could lead to variable antimicrobial activity against pathogens [156]. Furthermore, fractionation of the initial extract, using different solvents demonstrated variable antimicrobial effect on the same strain [163]. The correlation between used solvents and the exerted antimicrobial activity is extensively discussed in Section 5.

Variability of BCP and BB antimicrobial activity could be further attributed to implemented methods. Two main in vitro methods are used for this purpose. One is the broth dilution assay and the other is the agar well diffusion assay [164] or disc diffusion assay [155]. There are some factors that could affect the outcome of these assays. In broth dilution method there is an immediate and close contact between the active compounds contained in BCP or BB extracts and the target microorganism. On the contrary, in agar well diffusion assay, diffusion rates of active substances might be slower in agar than in broth. These active substances may not show the same diffusion rate in agar, due to different polarity, solubility, size or clogging tendency. Nevertheless, agar well diffusion in some cases might generate more reproducible data. In a study testing Chilean BCP extracts against *Streptococcus pyogenes* I.S.P. 364-00, BCB sampled for three consecutive years demonstrated the same average inhibition zone diameter in agar well diffusion assay but not the same minimum inhibitory concentration (MIC) values in broth dilution assay [126]. Reproducibility of agar well diffusion agar does not reflect higher sensitivity compared to broth dilution assay though. On the contrary, broth dilution assay is characterized by higher sensitivity.

Even if the implemented in vitro assay is the same, results are variable amongst different studies. One reason is that after evaporation of the initial solvent, the remaining dry matter is often dissolved in another solvent which is not the same in all studies. Such solvents might be dimethyl sulfoxide (DMSO) [47,166] or ultrapure water [73]. Therefore, one should take into account the particular chemical and physical effects of used solvents on the bacterial cell membrane system. These effects differ between bacterial species and strains [173].

In order to achieve best extraction of flavonoids, phenolic acids and fatty acids, the rigid pollen grain must be breached, disrupting exine and intine layers. Therefore, organic solvents, sonication and heat have been applied in combination. Such intense process may lead to a better outcome regarding antimicrobial and antioxidant activity, total phenolic or flavonoid concentration. Nevertheless, it is not evident whether BCP and BB polyphenols or other active compounds present in the different extracts are readily available to consumers through digestion of pollen grains. Humans cannot fully digest pollen grains, so it is estimated that nutrient bioavailability is reduced by 50% or more [37]. It is conceivable that extraction using water simulates better nutrient absorption during digestion in the human body. Furthermore, water extracts of BCP and BB are edible and ready to use as food ingredients.

## 5. Antimicrobial Activity of BCP and BB-Mοde of Action

Antimicrobial resistance is a global emerging threat so there is a great need for new antimicrobial products. Both BCP and BB demonstrate antimicrobial activity [46,47,50]. However, there is scarce literature on BCP and BB antimicrobial properties. Table 3 and Table 4 present studies on BCP and BB antimicrobial activity respectively. Moreover, they provide information regarding the geographical origin and the number of samples analyzed in different studies.

117 BCP samples and 20 BB samples were assessed for their antimicrobial activity in published studies. Most studies on BCP assess 1–5 samples. It is evident that a higher number of examined samples would offer a more detailed picture of the antimicrobial properties which are highly correlated with the chemical composition and therefore with botanical origin. Additionally, a higher number of conducted studies will identify new natural substances exerting targeted antimicrobial activity against certain pathogens.

Given that samples of diverse botanical origin or even the same botanical origin but extracted with different solvents exert variable activity, the lowest reported minimum inhibitory concentration (MIC) defines the most sensitive strain and the highest MIC the most resistant strains as depicted in Table 3 and Table 4. This comparison might define the optimum antimicrobial activity against certain pathogens among diverse samples (botanical/geographical origin) or different extraction methods. It seems that Gram-positive bacteria are in general, more susceptible to BCP [46,47,50,126,154,165,172] with some noticeable exceptions [156,163,166].

Nevertheless, in some studies, the same BCP sample may be more effective against either Gram–positive or Gram–negative bacteria depending on the used solvent during extraction, as previously mentioned. Authors reported that Gram–negative bacteria (*E. coli* and *C. jejuni*) were more sensitive against Slovenian BCP extracts (Table 3) than Gram–positive bacteria (*L. monocytogenes*) [166]. Also BCP extracts from Chile exhibited antibacterial activity against *S. pyogenes* I.S.P. 364-00 but didn’t exert any antibacterial activity against *E. coli* ATCC-25922, *S. aureus* ATCC-25923 and *P. aeruginosa* ATCC-27853 [126].

A study on Slovak BCP revealed that microbial strains showed variable susceptibility depending on the solvent used for the extraction of the BCP sample. *E. coli* CCM 3988 has been the most susceptible bacterial strain when tested against 70% ethanol extracts. On the contrary, against a 96% ethanol extract, this strain was much more resistant. *P. aeruginosa* CCM 1960, was the most resistant strain against all tested solvent extracts. These findings indicate that there is strain specificity depending on used solvent (e.g., methanol, ethanol) or concentration (e.g., 96% or 70% ethanol) [157]. The same finding was observed with *A. fumigatus*. This fungus was the most susceptible when tested against 70% ethanol extract amongst *Aspergillus* spp. but it was the most resistant when tested against 70% methanol extract.

In another study on bee collected *Trifolium alexanderinum* L. pollen, ethanol was used for the initial extraction followed by fractionation using solvents of increasing polarities, such as petroleum ether, dichloromethane and ethyl acetate. This procedure affected the phenolic content, as well as the antioxidant and antimicrobial activity of the extract. The initial ethanol extract, petroleum ether and dichloromethane fractions showed the highest antimicrobial activity against tested microorganisms while ethyl acetate showed the lowest antimicrobial activity. Nevertheless, ethyl acetate fraction demonstrated the highest phenolic and flavonoid content as well as the highest antioxidant activity alongside the ethanol extract [163]. These findings indicate that neither total phenolic/flavonoid content nor the antioxidant activity is directly proportional with antimicrobial activity.

In the same study, AbdElsalam et al. demonstrated that the initial 70% ethanol extract exhibited the highest antibacterial activity against *S. aureus* (38 mm inhibition zone), followed by *P. aeruginosa* (33 mm inhibition zone). In contrast the petroleum ether fraction showed the highest activity against *P. aeruginosa* (41 mm inhibition zone) followed by S. aureus (33 mm inhibition zone). It is noteworthy that petroleum ether fraction did not exert any activity against *A. niger* in contrast to other solvent fractions.

Although very few studies have been conducted regarding BB antimicrobial activity (Table 4), it seems that major findings are similar to that of BCP. For instance Gram-positive bacteria are more susceptible to BB than Gram-negative [35,172] though not always [50,168].

It is evident that there is significant variability regarding BCP and BB antimicrobial activity depending on botanical source, extraction procedure and tested microorganism. It should be noted that there are studies (Table 3) where antimicrobial activity was not detected at all [161], or moderately detected [126,165]. Therefore, a universal screening assay should be developed in order to optimize the activity against a specific pathogen, in a way analogous to an antibiogram.

Greek BCP extracts were reported to contain the following flavonoids: kaempferol 3-O-rhamnoside, quercetin 3-O-glucoside, quercetin 3-O-galactoside, quercetin 3-O-rhamnoside, isorhamnetin 3-O-xylosyl (1–6) glucoside, 7-Omethylherbacetin 3-O-sophoroside, and 7-O-methylherbacetin 3-O-glucosyl-8-O-galactoside. These molecules were used in pure form against Gram-positive and Gram-negative bacteria and inhibited both. However, lower MIC values were determined against Gram-positive bacteria [46].

The effect of BCP and BB extracts on host microbiome should be investigated in the future. Antibiotics are known to kill pathogens and probiotics indiscriminately. Interestingly, it has been reported that BCP exhibits antibacterial activity against pathogenic bacteria but not against lactic acid starter cultures [155]. Furthermore, quercetin aglycone, a BCP compound, did not exert any activity against *L. casei var shirota* [175]. Quercetin significantly improved the probiotic potential of broilers cecal microbiome. There was a decrease in numbers of Colony Forming Units (CFUs) of *P. aeruginosa*, *S. typhimurium*, *S. aureus*, and *E. coli* but significantly increased the numbers of CFUs of *Lactobacillus* spp., *Bifidobacterium* spp., and total bacteria [176]. It is plausible that BCP and BB compounds exert targeted activity against pathogens without disrupting the host microbiome but further research should be conducted.

Table 5 and Table 6 present BCP and BB activity against two major bacterial pathogens and some fungi, in a quantitative way. The MIC values range from 1–3 mg/mL to 2 μg/mL, which are in some cases similar or superior to that of antibiotics in clinical practice. For example, Moroccan BB extracts demonstrated superior MIC and Minimum Bactericidal Concentration (MBC) values compared to antibiotics against the tested pathogens [35]. The MBC values of Moroccan BB extracts against *S. typhimurium* (ATCC 13311) and *L. monocytogenes* (NCTC 7973) were 0.175 mg/mL for both bacteria. Ampicillin MBC values against *S. typhimurium* (ATCC 13311) and *L. monocytogenes* (NCTC 7973) were 1.20 mg/mL and 0.50 mg/mL respectively. In comparison streptomycin MBC values against *S. typhimurium* (ATCC 13311) and *L. monocytogenes* (NCTC 7973) were 0.30 mg/mL. Khider et al. compared the antimicrobial activity of maize (*Zea mays*), clover (*Trifolium alexandrinum*) and date palm (*Phoenix dactylifera*) BCP extracts to eleven antibiotics. Methanol crude extracts of maize and clover BCP exhibited inhibition activity (MIC values ranged from 320–1280 μg/mL and 320–640 μg/mL respectively) against pathogenic bacteria. This antibacterial activity was similar to antibiotics [155]. MIC values comparable to that of antibiotics were also reported for BCP extracts derived from the Greek endemic plant *Cistus creticus* L. (rock rose) [169].

Overall, *P. aeruginosa* is one of the most resistant bacteria against BCP and BB. In all cases the MIC values against *P. aeruginosa* were higher than those against *S. aureus*. Resistance exerted by Gram-negative bacteria might be attributed to cell wall structure which is more complex than that of Gram-positive bacteria [47,50,154,155]. Additionally, higher susceptibility of Gram-positive bacteria has been observed while testing BB samples from *Apis mellifera* and other bee species such as the stingless bee *Heterotrigona itama* [174].

In some studies antimicrobial activity is correlated with total phenolic content [177]. Other authors suggest that it is not the total phenolic content *per se* but its composition which correlates with antimicrobial activity. This is supported by the fact that BCP extracts with the lowest total phenol concentrations were the most effective against microorganisms [47,73,99].

Fatty acids such as linoleic, linolenic, myristic, and lauric, are also reported to exert bactericidal and antifungal properties [99]. Free fatty acids (FFAs) can be released from fatty acids or lipids primarily through enzyme activity. The antibacterial properties of FFAs are mainly attributed to the disruption of the electron transport chain and oxidative phosphorylation of the cell membrane. Furthermore, FFAs may also impair nutrient uptake, generate toxic peroxidation and cause direct lysis of bacterial cells [67]. The potential of FFAs as antibacterial agents is widely acknowledged [68].

The polyphenol compounds usually detected in BCP and their mode of antimicrobial action are presented in Table 7. Mode of action was elucidated using these compounds derived from plants or their synthetic forms, not BCB or BB crude extracts.

Quercetin and kaempherol (synthetic) tested against *C. parapsilosis* complex, inhibit biofilm formation and planktonic cell growth [178].

Similarly, luteolin exhibits antibacterial activity against planktonic cells and biofilm formation of three *S. aureus* and three *L. monocytogenes* strains [179].

Apigenin (synthetic) inhibits the enzyme d-alanine:d-alanine ligase [180], which catalyzes the production of the peptidoglycan precursor d-ala-d-ala, thus destabilizing the bacterial cell wall. Apigenin tested in synergy with other flavones reduced the production of *P. aeruginosa* virulence factors ciliotoxin and pyocyanin. On the contrary, there was a weak effect on growth of two coagulase-negative *Staphylococcus* (CNS) and two methicillin-resistant *S. aureus* (MRSA) clinical isolates. These data suggest that flavones may have anti-Gram-negative potential [181].

Galangin is an active constituent found in bee products such as BCP and propolis, and also in herbs such as *Helichrysum aureonitens*, traditionally used to treat infections. Galangin (synthetic) leads to significant potassium loss in *S. aureus* cells, which might be attributed to cytoplasmic membrane disruption [182]. Galangin was described to alter expression of cytochrome P450 isoenzymes in rats in long-term administration. This suggests that galangin could be implemented to enhance oral drug bioavailability and reverse multidrug resistance [183]. More studies should be conducted in the future to elucidate the effect of galangin or other flavonoids in antibiotic bioavailability.

Gallic acid and ferulic acid breach membrane integrity through hydrophobicity alteration, creating local membrane rupture or pore formation. Surprisingly, Gram–negative bacteria were more susceptible than Gram–positive ones. *P. aeruginosa* was the most susceptible bacterium to gallic acid, with MIC at 500 μg/mL. Together with *E. coli*, they were the most susceptible to ferulic acid with MIC at 100 μg/mL. Gram-positive bacteria were less susceptible to both phenolic acids with a MIC of 1750 μg/mL for gallic acid and at 1100 μg/mL for ferulic acid for *S. aureus*. For *L. monocytogenes* the MIC values were 2000 μg/mL and 1250 μg/mL for respectively [184].

Esters of caffeic acid inhibited bacterial growth of the bee pathogen *P. larvae* through an oxidative stress mechanism [185].

*p*-Coumaric acid has a dual mechanism of bactericidal activity disrupting bacterial cell membranes and binding to bacterial genomic DNA thus leading to cell death. *p*-coumaric acid significantly increased the membrane permeability, resulting in the loss of the barrier function. It was also demonstrated that *p*-coumaric acid could bind to the phosphate anion in the DNA double helix, and intercalate into DNA [186].

Bioavailability of phenolic compounds is an important factor in order to elucidate their antimicrobial action. Regarding flavonoids, absorption takes place in the small intestine and depends on several parameters. The highest bioavailability has been determined for isoflavones, followed by flavanols, flavanones and flavonol glycosides.

Flavonoid glycosides are deglycosylated prior to intestinal uptake, whereas aglycones can freely penetrate cell membranes. Afterwards, absorbed flavonoids are transported to the liver where they undergo extensive metabolism, thus generating different conjugation forms such as glucuronides, sulphates and methylated derivatives. These conjugates are responsible for the health-promoting effects of flavonoids [187]. Small amounts of flavonoids glycosides such as quercetin−3-*O*-glucoside, rutin and other quercetin glycosides are detected in the blood stream indicating a direct uptake mechanism [188,189]. It is reasonable to assume that deglycosylation process of flavonoids leads to aglycon products regardless of their origin (BCP or other plant parts). In the gastrointestinal tract a direct action of both glycosylated and aglycon forms might take place. This action could help to control intestinal infections caused by *H. pylori* and foodborne pathogens [180,190,191].

Other phenolic compounds, such as phenolic acids, are metabolized extensively after their absorption in the gastrointestinal tract. This includes methylation, glucuronidation and sulfation. Sulfation and glucuronidation negatively affects their antioxidant activity. There are limited data on the biological activity of phenolic acid metabolites. Phenolic acids exhibit antimicrobial activity and also act as food preservatives. The antimicrobial potential of phenolic acids depends on their chemical structure especially on the saturated chain length, position and number of substitutions in core benzene rings [192].

Recently, exosome-like vesicles (EXLVs) were detected in *Apis mellifera* hypopharyngeal gland secretions and in bee products (honey, royal jelly and bee pollen). EXLVs exerted bacteriostatic, bactericidal and biofilm-inhibiting effects on *S. aureus* [60].

Last but not least, there are indications that metabolites produced by BCP and BB microbiome contribute towards their antimicrobial activity. Antimicrobial activity has been determined in BCP inoculated with selected lactic acid bacteria and yeast strains, in a fermentation process simulating the conversion of BCP into BB. Inhibition against tested microorganisms was exerted after fermentation involving certain strains of *L. kunkeei* whereas the unfermented pollen extract showed no antimicrobial activity. This finding suggests that antimicrobial activity could be attributed to specific metabolites produced mainly by *L. kunkeei* strains [152]. In a very recent study after spontaneous BCP fermentation and fermentation with added *L. lactis* and *L. rhamnosus*, total phenolic and flavonoid content as well as radical scavenging activity increased by 1.27–2.40 fold, antibacterial activity against *M. luteus*, *S. aureus* and *E. coli* increased by 1.08–16.9 fold and antifungal activity against *P. roqueforti* increased by 1.96–5.52 fold after bee pollen fermentation [48]. These studies clearly suggest that the antimicrobial activity exerted by BB could be attributed not only to pollen phytochemicals but also to metabolites produced by BCP and BB microbiome. Nevertheless, this has to be confirmed in future studies.

BCP and BB potentially exhibit antiviral activity. However, current studies focus on the antiviral activity of BCP compounds and not BCP or BB extracts. Flavonoids and one alkaloid found in Korean *Papaver rhoeas* BCP exhibit neuraminidase inhibitory activity against influenza strains H1N1, H3N2, and H5N1. Tested flavonoids were kaempferol-3-sophoroside, kaempferol-3-neohesperidoside, kaempferol-3-sambubioside, kaempferol-3-glucoside, quercetin-3-sophoroside, luteolin, and chelianthifoline. All compounds have shown neuraminidase inhibitory activities with IC_50_ values ranging from 10.7 to 151.1 µM. The most potent neuraminidase inhibitor was luteolin, which was the dominant flavonoid in the ethyl acetate fraction [69]. Extracts from date palm pollen exhibited antiviral activity against vesicular stomatitis virus which was further increased after fermentation using *Trichoderma koningii*. Pollen fermentation improved unsaturated fatty acid ester and flavonoid production [193]. Flavonoids have shown activity against Herpes Simplex Virus-1 (HSV-1) and Herpes Simplex Virus-2 (HSV-2). Among flavonols, galangin, and kaempferol demonstrated the most potent antiviral activity [194]. Galangin isolated from the shoots of *Helichrysum aureonitens* showed significant antiviral activity against HSV-1 and Coxsackie B1 [195]. Additionally, quercetin has demonstrated binding affinity to influenza HA protein and inhibited the entry of H5N1 virus in host cells [196].

There is research interest regarding interaction of flavonoids with viral proteases of SARS- and MERS-CoV [197,198]. Interestingly, quercetin, kaempherol and their glycoside derivatives which are present at high concentration in BCP, may exhibit antiviral activity against SARS-CoV-2 and MERS [199]. Quercetin and its derivatives exhibit in vitro antiviral activity against several viruses, inhibiting viral DNA and RNA polymerases or bind to essential viral proteins [200]. Quercetin-3β-galactoside, binds to SARS-CoV chymotrypsin-like protease (3CLpro), at Gln189 residue, blocking its proteolytic activity [201]. SARS-CoV-2 3CLpro is very similar to that of SARS-CoV and maintains the same residue in its active site [202], so might also be sensitive to the inhibitory action of quercetin and its derivatives. Interestingly, we aligned 3CLpro protein sequences from publicly available Beta coronavirus genomes from all 5 subgenera and observed that this residue (Gln 189) was 100% conserved in all of them [Amoutzias unpublished data]. The 3a protein forms one of the three putative ion channels of SARS-CoV-2 which is expressed in the infected cell and it is involved in virus release [203]. Kaempferol and its glycoside analogs inhibit the 3a channel protein of coronavirus [204].

Taking into account all available data, total phenolic content and phenol composition are two major factors directly related to antimicrobial activity exerted by BCP extracts. There are other antimicrobial factors, such as fatty acids, EXLVs and presumably microbial metabolites. Phytosterols could act in synergy enhancing the antimicrobial properties of the above-mentioned factors [205]. Although, there are very few studies measuring the concentration of these active substances in BCP or BB, it is possible that compounds present in pollen will be present in bee collected pollen and bee bread as well, but their concentration still needs to be further studied.

Several studies have demonstrated that BCP compounds can act synergistically against pathogens [46,155,209]. Moreover, it is suggested that BCP extracts or specific BCP compounds can exert antimicrobial activity in synergy with antibiotics [154]. This has been tested for kaempferol glycosides of plant origin in synergy with hydrophilic fluoroquinolones against methicillin-resistant *S. aureus* (MRSA). Kaempferol greatly reduced the MICs of the implemented antibiotic [206]. On the other hand, apigenin used in combination with other flavones did not enhance the antimicrobial activity of a penicillin/streptomycin mix against coagulase-negative *Staphylococcus* (CNS) and two MRSA strains. In the same study synergy was observed against *P. aeruginosa* in the presence of antibiotics or recombinant human lysozyme [181]. In order to choose appropriate BCP or BB extracts or their constituents that could be implemented in combination with certain antibiotics, a standardized protocol should be developed. Synergy of BCP and BB with antibiotics could prevent or delay antimicrobial resistance [206]. There is no report on microorganisms that have developed resistance to a combination of compounds present in BCP or BB until now, but this should be confirmed in future studies. There is an urgent need for new nontoxic and efficient antifungal agents. In that respect honeybee products and, particularly propolis and pollen, can help to control fluconazole-resistant fungal strains [170].

## 6. Conclusions and Future Perspectives

It is evident that BCP and BB are promising antimicrobial agents to control multidrug resistant bacteria and other pathogens including viruses.

However, antimicrobial properties exerted by BCP and BB are highly variable. This variability could be attributed to differences in chemical composition which directly correlates to pollen botanical origin. Phytochemical concentrations, including flavonoids, phenolic acids, fatty acids and phytosterols vary significantly among different plant species.

Given the enormous plant variety in a wide range of habitats where honeybees collect pollen, it is obvious that a tiny portion of this biodiversity has been tested so far. More studies should be conducted all over the world, aiming to bioprospect novel antimicrobial compounds present in BCP and BB derived from medicinal and endemic plant species.

Data comparison and interpretation of results among different studies which focus on BCP and BB antimicrobial activity is not easy sometimes, due to different methodologies. Sampling is important, especially when samples are not taken directly from the hive but from commercially available products. Storage conditions may deteriorate sample quality. Furthermore, extraction procedures using various solvents and protocols might lead to hard to compare data of BCP and BB samples even if they are derived from the same plants. Therefore, standard operating procedures followed by researchers in the field should be developed not only regarding extraction protocols but bioassays too.

For instance, it is acknowledged that broth dilution assay is more sensitive compared to agar well diffusion assay. Therefore, it could be more appropriate to implement this method in order to get comparable data.

Although there is high variability of BCP and BB antimicrobial activity, the following general conclusion can be drawn: BCP or BB demonstrate selective antimicrobial activity that is usually higher against Gram-positive compared to Gram-negative bacteria. Strain specificity should be further investigated to identify BCP/BB compounds responsible for that. In that respect more studies should be conducted towards exploring synergy of BCP and BB extracts (or compounds detected within) and antibiotics. This synergy could prevent or delay microbial resistance which is a major public health issue.

Regarding the mode of action, there is circumstantial evidence based on compounds derived mostly from plants. It is clear that more intensive research to elucidate the antimicrobial molecular mechanisms is needed. Cutting-edge OMICS technologies like RNA-sequencing should be implemented to identify at whole transcriptome level, the molecular targets of BCP/BB crude extracts (or specific compounds detected within) in different pathogens.

Another OMICS technology, next generation sequencing (NGS) has already been implemented to study the BCP and BB microbiome. Microbial communities differ significantly among BCP and BB. LABs play a pivotal role in BCP fermentation to BB, though yeasts apparently have an important role too. Whether pre-digestion of pollen grains by BB microbiota takes place is still controversial. It is plausible that BCP and BB microbiome produce compounds (f.i. bacteriocins) that exert antimicrobial activity. Once again direct evidence is still lacking. Future research should further explore BCP/BB microbiome not only as a source of antimicrobials, but also as an authenticity and quality biomarker (for instance high relative abundance of LABs could confer probiotic potential). NGS technologies have dropped dramatically the cost of sequencing and at the same time increased exponentially our technical capacity to obtain genomic datasets. Therefore, extensive NGS implementation on BCP and BB samples from diverse geographical and botanical origin might lead to unique microbial fingerprints useful as authenticity biomarkers.

Another important research direction should focus on BCP and BB effects on human gut microbiome and their prebiotic potential. Detailed studies on ingredient assimilation and bioavailability should be conducted thus further assessing BCP and BB effects.

Nowadays, consumers are increasingly keen towards superfoods and functional foods, due to high nutritional value as well as human well-being and health promotion. There is no doubt that BCP and BB could be considered as superfoods. Nevertheless, intensive research is still necessary before hard evidence supports health claims and applications of BCP and BB in clinical practice as a source of antimicrobials.

## Figures and Tables

**Table 1:** Bee product compounds that exhibit antimicrobial activity.

Honey	Propolis	Royal Jelly	Pollen	Beebread	Bee Venom
H_2_O_2_ [51,52]		Glucose oxidase [53]			
Methyl glyoxal [54]					
MRJPs [55,56]		MRJPs [55]			
Bee Defensin-1 (Royalisin) [6,57]	AMPs [58]	Bee defensin-1 (Royalisin) [59]			
Ex.L.V [60]		Ex.L.V [60]	Ex.L.V [60]		
		Jeleins [61]			
Polyphenols [62,63]	Polyphenols [62,63]	Polyphenols [64]	Polyphenols [62,63]	Polyphenols [62,63]	
		Hydroxydecenoic acid derivatives (10-HDA fatty acid) [65,66]	Fatty acids [67,68]	Fatty acids [67,68]	
1,2-Dicarbonyls [6]					
			Alkaloids [69]		Melitin Phospholipase A2 [29]

(AMPs: antimicrobial peptides), (Ex.L.V: exosome-like vesicles), (MRJPs: Major Royal Jelly Proteins).

**Table 2:** Different solvents used for BCP and BB samples extraction.

Solvent Extract	BCP	Bee Bread
DMSO	[50]	[50]
Methanol	[46,47,154,155,156,157,158,159,160,161]	[35]
Ethanol	[126,156,157,162,163,164,165,166,167]	[168]
Boutanol	[169]	
Dicloromethane	[46,169]	
Hexane	[155,169]	
* Water	[46,73,169,170,171]	[49,172]

* deionized or distilled.

**Table 3:** Antimicrobial activity of BCP samples from different countries.

Origin of Samples	Number of Samples	Most Susceptible Bacteria Strains According to Sample and Extraction Method	Most Resistant Bacteria Strains According to Sample and Extraction Method	Most Susceptible Fungus and Yeast Strains According to Sample and Extraction Method	Most Resistant Fungus and Yeast Strains According to Sample and Extraction Method
Morocco [50]	4	*-S. aureus* (r) *Streptococcus spp* (r)	*-P. aeruginosa* (r) -*E. coli* (r)		
Greece [46]	1com	*S. aureus* (ATCC 25923)	*E. coli* (ATCC 25922)	*-C. glabrata* (ATCC 28838) methanol extract *-C. tropicalis* (ATCC 13801) aqueous extract	*C. albicans* (ATCC 10231) methanol extract
Greece [169]	3 (1mono)	*S. epidermidis* (ATCC 12228)	*E. cloacae* (ATCC 13047)	*C. glabrata* (ATCC 28838)	*C. albicans* (ATCC 10231)
Portugal and Spain [154]	8	*S. aureus* (ATCC 6538™)	*E. coli* (ESA37)	*C. glabrata* (ATCC 66032^TM^)	*C. glabrata* (ESA 123)
Portugal [47]	5	*B. cereus* (ESA 55)	*E. coli* (ESA 15)	*Z. bailii* (ESA 1307)	*C. magnoliae* (ESA 11)
Egypt [163]	1	*-S. aureus* ethanol extract *-P. aeruginosa* pet. ether and DCM fraction	*-S. aureus* pet. ether and DCM fraction *-P. aeruginosa* ethanol extract	*A. niger*	*C. albicans*
Egypt [155]	3 mono	*S. aureus* (ATCC 8095)	*P. aeruginosa*		
Egypt [158]	1mono	*L. monocytogenes* (CIP 82.110)	*-S. enterica* (CIP 81.32)		
Turkey [162]	5			* A. parasiticus (NRRL 2998)	
Turkey [170]	1			*C. albicans*	*-C. krusei* *-Trichosporon* spp.
Turkey [161]	1com	nd^+^	nd^+^	nd^+^	nd^+^
Turkey [160]	9	*S. aureus* MRSA	*K. pneumoniae* nd	*C. krusei* (ATCC 6258)	*C. albicans* (ATCC 14053)
Turkey [165]	5	*L. monocytogenes* (ATCC 15313) *S. aureus* (ATCC 29213)	*E. coli O157:H7* (NCTC 12900) nd *S.* enteritidis (ATCC 13311) nd		
Turkey [159]	5				* A. alternata * F. oxysporium
Slovakia [156]	3 mono	*S. enterica* (CCM 4420) *S. aureus* (CCM 3953)	*P. aeruginosa* (CCM 1960) *L. monocytogenes* (CCM 4699)		
Slovakia [157]	1com	*E. coli* (CCM 3988) 70% ethanol	*P. aeruginosa* (CCM 1960)	*A. fumigatus* 70% ethanol *C. glabrata* 70% methanol	*A. flavus* *A. fumigatus* 70% methanol *C. krusei* 99.9% methanol and 70% ethanol
Slovakia [164]	1com	*C. butyricum* *C. perfringens*	*C. intestinale*		
Slovenia [166]	14	*E. coli* *C. jejuni*	*L. monocytogenes* nd		
Chile [126]	29	*S. Pyogenes* (I.S.P. 364-00)	*E. coli* (ATCC-25922) nd *S. aureus* (ATCC-25923) nd *P. aeruginosa* (ATCC-27853) nd		
Chile [73]	16	*S. aureus* (ATCC-25923) *-S. pyogenes* (I.S.P. 364-00)	*E. coli* (ATCC-25922) *P. aeruginosa* (ATCC 27853)		
Chile [171]	1	*S. aureus* *S. pyogenes*	*E. coli* *P. aeruginosa*		

nd: antimicrobial activity was not detected nd^+^: antimicrobial activity was not detected against tested microorganisms: *B. cereus*, *B. subtilis*, *E. coli*, *S. typhimurium*, *S. aureus*, *Y. enterocolitica*, *E. faecalis*, *L. monocytogenes*, *S. cerevisiae*, *C. rugose*, *A. niger* and *R. oryzae* [161]. Com: combined samples of the same geographical origin. mono: high percentage monofloral sample. r: antibiotic resistant strain. ATCC: American Type Culture Collection. NCTC: National Collection of Type Cultures. ESA: Escola Superior Agraria de Braganca. CIP: Collection of Institut Pasteur. NRLL: Agricultural Research Service Culture Collection. CCM: Czech Collection of Microorganisms. ISP: International Cooperative Project for Description and Deposition of Type Cultures of Streptomyces. *: only one or two strains were used in this study. The solvent used in the extraction method is only referred when affecting the susceptibility and/or the resistance. More than one bacteria or fungi are reported in case that there are minor differences in susceptibility.

**Table 4:** Antimicrobial activity of BB samples from different countries.

Geographic Origin	Number of Samples	Most Susceptible Bacteria Strains According to Sample and Extraction Method	Most Resistant Bacteria Strains According to Sample and Extraction Method	Most Susceptible Fungus and Yeasts Strains According to Sample and Extraction Method	Most Resistant Fungus and Yeast Strains According to Sample and Extraction Method
Morocco [50]	4	*S. aureus* 2 (r) sample3,4 *E. coli* 3 (r) sample3 *S. aureus* (ATCC25923) (r) sample3 *B. cereus* sample2	*B. cereus* sample1 *P. aeruginosa* (ATCC29733) (r) sample1 *E. coli* 2 (r) sample2,3		
Morocco [35]	1	*B. cereus* (food isolate)	*E. coli* (ATCC 35210)	*A. ochraceus* (ATCC 12066)	*-A. niger* (ATCC 6275) *-P. ochrochloron* (ATCC 9112) *-P. cyclopium* (food isolate)
Lithuania [49]	4	*S. aureus*	*S. epidermidis*		
Czech Republic [167]	4	*S. sobrinus*	*S. mutans*		
Romania [172]	1	*S. aureus*	*E. coli*		
Malaysia [174]	1	*S. aureus* *B. cereus*	*E. coli* *Salmonela spp.*		
Ukraine [168]	5	*E. coli* (CCM 3988) *S. enterica* (CCM 3807)	*S. aureus* (CCM 4223) *B. thuringiensis* (CCM 19)		

r: antibiotic resistant strain. Given that samples of diverse botanical origin or even the same botanical origin but extracted with different solvents exert variable activity, the lower reported Minimum Inhibitory Concentration (MIC) defines the most sensitive strain and the higher MIC the most resistant (in some cases are the same). More than one bacteria or fungi are reported in case that there are minor differences in susceptibility.

**Table 5:** MIC values (mg/mL) of BCP and BB against different *S. aureus* and *P. aeruginosa* strains.

Microorganism Strain	MIC
*S. aureus* (ATCC 6538^TM^)	BCP: 1.81 [154]
*S. aureus* (ESA 159)	BCP: 2.58 [154]
*S. aureus* (ATCC 25923)	BCP: 0.5 [46]
*S. aureus* (ATCC 25923)	BCP: 2 × 10^−3^ [169]
*S. aureus* (ATCC 8095)	BCP: 0.32 [155]
*S. aureus* (CIP 76.25)	BCP: 0.78 [158]
*S. aureus* (ATCC 6538)	BB: 0.175 [35]
*P. aeruginosa* (ATCC™)	BCP: 3.71 [154]
*P. aeruginosa* (ATCC 227853)	BCP: 1.35 [46]
*P. aeruginosa* (ATCC 227853)	BCP: 2.47 × 10^−3^ [169]
*P. aeruginosa* (PAO1)	BCP: 0.64 [155]

**Table 6:** MIC values (mg/mL) of BCP and BB samples against fungi and yeasts strains.

Microorganism Strain	BCP MIC	Bee Bread MIC
*C. albicans* (ATCC 10231)	4.81 [46]	
*C. albicans* (ATCC 10231)	3.34 × 10^−3^ [169]	
*C. albicans*	0.015 × 10^−3^ (24 h) [170]	
*C. glabrata* (ATCC 28838)	3.22 [46]	
*C. glabrata* (ATCC 66032^TM^)	16.00 [154]	
*C. glabrata* (ESA 123)	22.67 [154]	
*C. glabrata* (ATCC 28838)	3.14 × 10^−3^ [169]	
*C. glabrata*	0.0625 × 10^−3^ (24 h) [170]	
*C. tropicalis* (ATCC 13801)	3.00 [46]	
*C. tropicalis* (ATCC 13801)	3.20 × 10^−3^ [169]	
*C. krusei*	0.0075 × 10^−3^ (24 h) [170]	
*Trichosporon spp.*	0.002 × 10^−3^ (24 h) [170]	
*A. fumigatus* (ATCC 1022)		0.50 [35]
*A. ochraceus* (ATCC 12066)		0.35 [35]
*A. niger* (ATCC 6275)		1 [35]
*P. funiculosum* (ATCC 36839)		0.70 [35]
*P. ochrochloron* (ATCC 9112)		1 [35]
*P. cyclopium* (food isolate)		1 [35]

**Table 7:** Antimicrobial compounds present in BCP and their mode of action.

Compounds		Mechanism
Flavonoids	Quercetin glycosides	Damage bacterial cell wall and membrane, affect transport and motility [176]. Yeast and fungus biofilm control [178].
	Kaempferol glycosides	Yeast and fungus biofilm control [178] Inhibition of topoisomerase IV [206].
	Myricetin	Inhibits *E. coli* DnaB helicase [207].
	Luteolin	Impairing bacterial cell membranes, antibiofilm activities [179].
	Apigenin	Destabilizing cell wall components [180,181].
	Galangin	Bacterial cells aggregation [208]. Bacterial cells cytoplasmic membrane damage, potassium loss [182].
Other phenolic compounds	Ferulic acid	Rupture bacterial cell membranes, alterations in surface hydrophobicity [184].
	Gallic acid	Rupture bacterial cell membranes, alterations in surface hydrophobicity [184].
	esters of caffeic acid	Inhibits bacterial growth through an oxidative stress mechanism [185].
	*p*-Coumaric acid	Disrupts bacterial cell membranes and binds to bacterial genomic DNA [186].
